# National immunisation recommendations: why country-specific evidence and context matter

**DOI:** 10.1016/j.lanepe.2026.101835

**Published:** 2026-08-29

**Authors:** Robin Schaefer, Thomas Harder, Frank Sandmann, Ole Wichmann

**Affiliations:** Immunization Unit, Robert Koch Institute, Berlin, Germany

**Keywords:** Vaccines, Immunisation, Evidence-based recommendations, Country-specific evidence, National Immunisation Technical Advisory Groups

## Abstract

National Immunisation Technical Advisory Groups (NITAGs) are independent expert groups issuing recommendations on the introduction and use of licensed vaccines in national programmes. They consider both context-free evidence — safety, efficacy, effectiveness — and country-specific aspects, including local epidemiology, cost-effectiveness, societal values, and programmatic feasibility. Several policy developments in the European Union (EU), including EU-level joint clinical assessments under the new Health Technology Assessment regulation and non-binding recommendations by the European Centre for Disease Prevention and Control, reflect a desire to reduce duplication of efforts but also a push towards harmonisation of immunisation recommendations across countries. Using the German NITAG as an example, we illustrate that identical clinical evidence can legitimately yield different national recommendations. Even though equitable access to vaccines across countries is desirable, fundamental differences in country-specific context limit harmonising national immunisation schedules. International collaboration and supra-national guidance can support but not substitute for national, context-sensitive public health decision-making.

## Introduction

Vaccines are one of the most impactful and cost-effective public health interventions. Regulatory authorities, such as the European Medicines Agency (EMA) in the European Union (EU), base decisions of licensure of vaccines on a review of the quality, safety, and efficacy of the product. National Immunisation Technical Advisory Groups (NITAGs) evaluate as independent expert groups whether, how, and for whom a licensed vaccine should be used in a given population. In most EU countries, the ministry of health alone or together with other stakeholders (e.g., ministry of finance or regional authorities) considers the recommendations of the NITAG and makes the final decision on the introduction of a new vaccine in the publicly funded national immunisation programme.^1^ A NITAG enables national authorities to make independent, balanced, and evidence-based decisions and adds credibility to the immunisation programme.^2^

The processes of NITAGs differ between countries. However, all NITAGs consider both context-free evidence (e.g., vaccine efficacy, effectiveness and safety) and context-specific evidence (e.g., local disease epidemiology, cost-effectiveness, or programmatic issues).^1^ These evidence assessments are time- and labour-intensive. Therefore, to avoid duplication of efforts and to make better use of scarce resources, EU member states collaborate within the EU NITAG collaboration on the assessment of context-free evidence by jointly conducting systematic literature reviews and sharing best practices. This collaboration was initiated in 2018 and is coordinated by the European Centre for Disease Prevention and Control (ECDC).^3^

While these collaborative efforts are strictly limited to the assessment of context-free evidence, several recent policy developments in the EU reflect a growing push towards harmonisation of immunisation recommendations across countries. In 2021, the new regulation on Health Technology Assessment (HTA) was adopted in the EU, which came into force in 2025.^4^ Generally, HTA is a multidisciplinary, evidence-based process that evaluates the safety, effectiveness, efficiency, and social and ethical impact of health technologies in comparison to current or other new technologies. Starting with oncology drugs, the new EU regulation requires a joint clinical assessment (JCA) at EU level. New vaccines are required to undergo the JCA starting in 2030. The JCA reports will describe the health problem, safety and clinical efficacy of the health technology, and the certainty of evidence. However, they will not include the assessment of context-specific aspects, such as health economic and health system considerations, which will remain a matter of national competence in accordance with Article 168 of the Treaty on the Functioning of the EU.^5^ It will furthermore only apply to new immunisation products, not already licensed ones. Beyond HTA, an updated EU regulation that came into force in 2022 amended the mandate of the ECDC to allow for issuing of non-binding, evidence-based recommendations, including on immunisation strategies.^6^

Differences between national vaccination schedules in the EU exist and have been cited as a justification for streamlining national processes for assessing immunisation products and to support national authorities in making more informed and timely decisions, with the ultimate goal of accelerating equitable population access to vaccinations across EU member states.^7^^,^^8^ Importantly, however, vaccination schedules do not usually differ between countries because of concerns regarding context-free evidence or different interpretation thereof. NITAGs generally only issue recommendations for licensed vaccines that have already been approved by regulatory authorities and are thus demonstrated to be safe and effective. Instead, differences between national vaccination schedules commonly reflect country-specific considerations and context.

While both initiatives, the JCA at EU-level and EU-wide vaccination recommendations developed by ECDC, are designed to support, rather than replace, national decision-making, the scope of these activities, the framing of supra-national evidence assessments and recommendations, and their integration with existing national processes — including the role of NITAGs — will critically shape whether they reinforce or constrain country-driven decision-making.

Using the Standing Committee on Vaccination (STIKO), the German NITAG, as an example, we illustrate how national immunisation recommendation-making builds upon context-free and country-specific evidence and considerations. We argue that efforts to harmonise vaccination schedules across countries or to develop supra-national vaccination strategies face inherent limits, given country-specific particularities.

## Immunisation recommendation decision-making and the STIKO operating procedures

STIKO consists of 16–22 members, who are appointed by the German Federal Ministry of Health based on expertise across relevant disciplines, such as paediatrics, general medicine, immunology, epidemiology, public health, and infectious disease modelling. The committee is supported by an executive secretariat at the Robert Koch Institute, the national public health institute in Germany, with dedicated in-house capacity in epidemiology, mathematical modelling, and evidence synthesis. The level of institutional capacity and support is more extensive than that available to many other European NITAGs, and STIKO is therefore not fully representative of the European landscape in this respect. To determine its agenda, STIKO implements a prioritisation process for the evaluation of new vaccines and other immunisation products (such as long-acting monoclonal antibodies) or updating existing recommendations. This is also informed by a horizon scanning process to identify new products in the development pipeline.

**Fig. 1:**
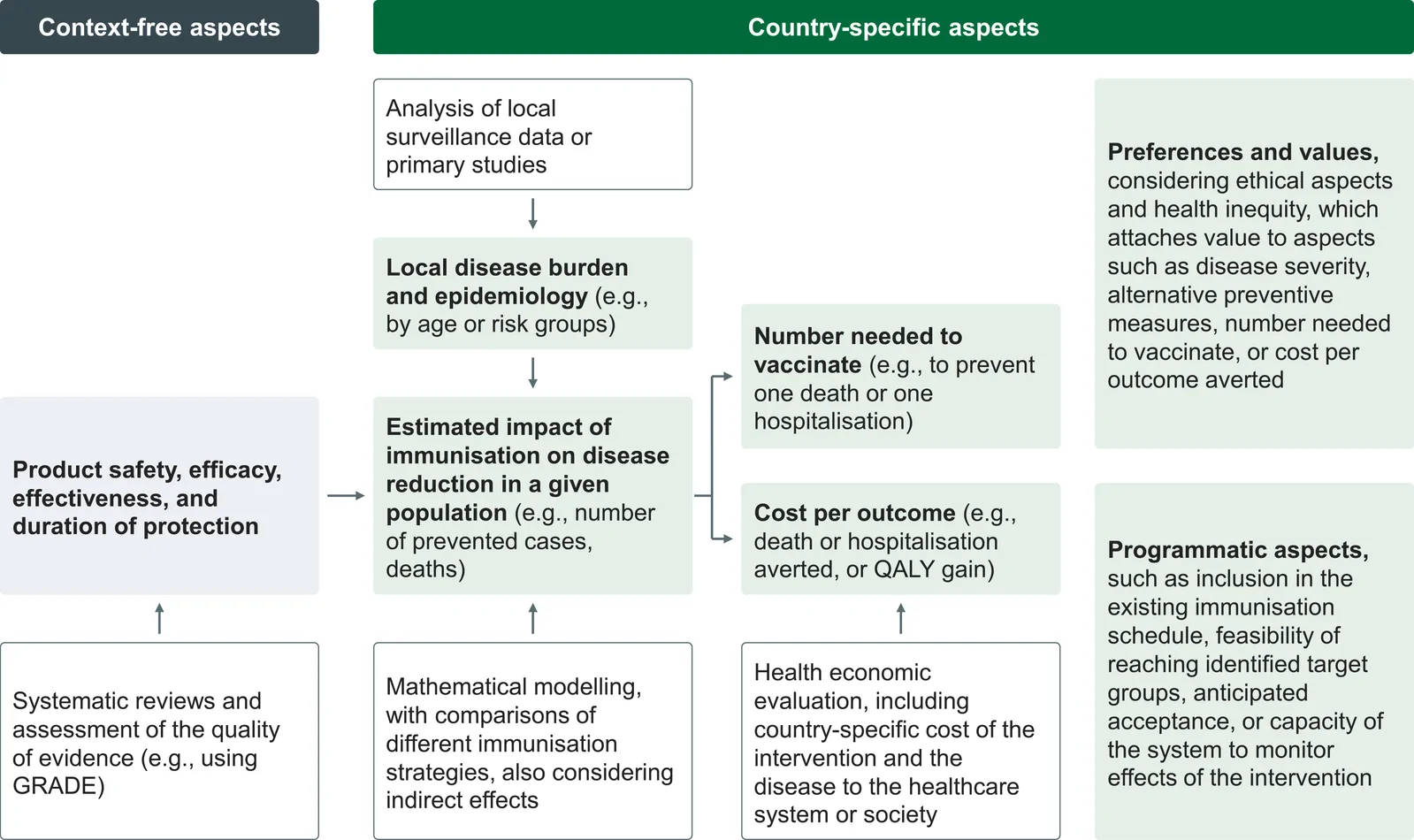
Aspects considered by the Standing Committee on Vaccination (STIKO) in Germany during the development of immunisation recommendations. Shaded boxes indicate the aspects considered in the assessment; white boxes indicate the underlying evidence and methods used to inform them. GRADE: Grading of Recommendations, Assessment, Development, and Evaluation; QALY: quality-adjusted life year.

STIKO follows a standard operating procedure (SOP) for assessing evidence and formulating recommendations.^9^ In line with international standards and broadly aligned with processes implemented by the Strategic Advisory Group of Experts on Immunization (SAGE) of the World Health Organization (WHO) and the practices of other European NITAGs,^1^ an Evidence-to-Decision (EtD) framework is used for synthesising and assessing evidence. The EtD framework provides a common structure for integrating diverse types of evidence within a single deliberation. Context-free evidence enters this framework primarily through the assessment of the safety, efficacy, and effectiveness of vaccines. Context-specific evidence informs almost every other domain, including the public health relevance, benefits and harms of vaccination at the population level, the feasibility and acceptability of implementation, as well as ethical and health economic considerations. Fig. 1 illustrates STIKO's decision-making process, highlighting the distinction between context-free evidence and context-specific considerations. The framework therefore makes clear that assessing safety and efficacy is a necessary but not sufficient basis for a national recommendation.

To develop recommendations, STIKO establishes topic-specific working groups composed of STIKO members and, possibly, external experts, who are carefully assessed for potential conflicts of interests. The working group prepares a draft recommendation and scientific rationale, which is deliberated and formally voted on by STIKO. During plenary deliberation, the committee assesses how the evidence in each EtD domain bears on the recommendation under consideration. The relative weight given to context-free and context-specific evidence is arrived at through structured discussion of the EtD domains, with explicit judgements recorded for each. Following the plenary vote on the draft, a non-public consultation is conducted with relevant stakeholders, including the federal states of Germany, professional societies and the Federal Joint Committee, which determines coverage by statutory health insurances. The final recommendation is subsequently adopted by a further formal vote by STIKO and published together with the full scientific rationale. The published rationale documents the evidence considered in each domain of the EtD framework, the weighting of context-free and context-specific considerations, and the reasoning that led to the final recommendation, making the weighting transparent and open to scrutiny. After the STIKO recommendation has been published, the Federal Joint Committee (i.e., the highest decision-making body of the joint self-government of physicians, dentists, hospitals and health insurance funds in Germany) has two months to incorporate the vaccine, based on the STIKO recommendation, into the national vaccination directive, which obliges statutory health insurances to cover them. Decisions on the financing of recommended vaccinations therefore follow relatively quickly after the endorsement of the STIKO recommendation and do not involve the ministry of health or ministry of finance, a feature specific to the German healthcare system.

## Context-free evidence: shared scientific foundations

According to its SOP, when considering a new vaccine recommendation, STIKO evaluates vaccine safety, efficacy and effectiveness against relevant clinical endpoints by performing systematic literature reviews and assessing the quality of evidence by applying the GRADE approach (Grading of Recommendations, Assessment, Development, and Evaluation).^9^ STIKO considers evidence from randomised controlled trials and observational (real-world evidence) studies. If a vaccine is already on the market targeting the same disease, data on relative safety and effectiveness (i.e., using the other vaccine as comparator) are considered.

This type of evidence is largely independent of local epidemiology or health system characteristics. The synthesis of this evidence therefore lends itself to international collaboration. This can reduce duplication of efforts and promote consistency in the interpretation of scientific data, providing a common scientific baseline for immunisation policymaking across countries.

## Context-specific considerations: the drivers of recommendations

Robust and high-certainty evidence of vaccine safety and efficacy does not, on its own, determine the appropriateness of vaccination in a given population. In fact, country-specific evidence and considerations are central to immunisation recommendation-making, and they explain why recommendations may differ between countries despite identical context-free evidence. These factors encompass several interrelated domains, which are considered together. Their cumulative balance can shift a positive context-free assessment into a recommendation for the general population, a recommendation only for specific risk groups, or no recommendation at all. STIKO considers the following aspects:

(i) **Local epidemiology and burden of disease:** This includes incidence, prevalence, the distribution of cases (e.g., by age and risk groups), severity of disease, and the size of risk groups. While intrinsic disease severity is unlikely to vary substantially between countries, observed population-level outcomes (e.g., case-fatality and hospitalisation rates) may differ substantially depending on age structure, prevalence of comorbidities, and access to care. These considerations determine the public health relevance of the disease, which influences whether a new vaccine or existing recommendation is evaluated by the NITAG. They may also inform whether a general recommendation is issued, one for specific risk groups, or none at all.
(ii) **Potential public health impact of the intervention:** This includes population-level desirable and undesirable effects (including herd protection and serotype replacement, e.g., for pneumococcal disease). Again, local epidemiology is central to this assessment, and besides robust epidemiological studies and surveillance activities, mathematical modelling studies may be required to understand the effects of different immunisation strategies, including the status quo or the adaptation of an existing recommendation. These modelling studies commonly require a number of strongly context-specific input parameters, limiting the utility of regional (e.g., EU-wide) models for NITAG decision-making, given that these are necessarily less specific than country-specific models.
(iii) **Health economic considerations:** This may include estimates of the number needed to vaccinate to prevent one relevant outcome (e.g., hospitalisation and death), as well as cost-effectiveness and cost-utility measures such as the additional costs per gain in quality-adjusted life years (QALYs). Mathematical modelling studies and health economic evaluations are needed to robustly estimate these measures. As costs of vaccination programmes, costs of the disease to the healthcare system and society, healthcare utilisation patterns, and budgetary constraints differ between countries, the relative value of a vaccination may vary fundamentally across countries. NITAGs may also differ in the weight they assign to health economic considerations in decision-making.
(iv) **Societal values and preferences and ethical considerations:** This includes how disease severity and different outcomes are weighted by societies, how benefits and risks are distributed across population groups, and how equity considerations are addressed. For example, protecting vulnerable populations or reducing health inequities may be prioritised differently depending on national contexts. It also involves preferences and values of healthcare providers, for instance regarding administering more than two vaccine doses during one visit or the off-label use of vaccines (which has liability implications in some countries, e.g., in Germany).
(v) **Programmatic and overall health system factors:** These include the feasibility of integrating a vaccine into existing schedules and reaching target groups, anticipated acceptance by healthcare providers and the public, alternative preventive measures and whether access to such measures exists (e.g., where universal health coverage is available), and the ability to monitor vaccine uptake, impact, and safety of the programme.

## Same clinical evidence, different conclusions

Despite identical context-free evidence, country-specific factors might lead to different, yet equally evidence-based recommendations. For example, a vaccine with high effectiveness against a disease of low incidence or low mortality may not be recommended for routine use in one country, but it may be prioritised in another country, in which the disease burden is higher. Different priority can also be given to the prevention of diseases that do not cause high mortality but result in substantial numbers of hospitalisations (e.g., rotavirus infection in many high-income countries) or loss of quality of life (e.g., herpes zoster). Similarly, different assessments of cost-effectiveness or programmatic feasibility can result in variations in target groups, the age at vaccination, or even the number and timing of vaccine doses. Table 1 provides examples of vaccine recommendations by STIKO and critical drivers of decision-making that include a range of country-specific considerations.

**Table 1:** Examples of recommendations of the Standing Committee on Vaccination (STIKO) in Germany and critical context-specific drivers.

Topic	STIKO recommendation	Year	Driver for decision-making^a^	References
Meningococcal disease (serotypes A, C, W, Y)	Removal of recommendation of vaccination against Meningococcal C among infants; recommendation of vaccination against Meningococcal ACWY among adolescents	2025	• Changing epidemiology and disease burden (decreasing incidence of Meningococcal C over many years, resulting in a very low burden of disease from Meningococcal C); epidemiological data on disease burden by serotypes • Mathematical modelling of the impact and efficiency of different immunisation strategies, including removing the recommendation to vaccinate infants against Meningococcal C in Germany and the switch to a quadrivalent vaccine • Simplification of the childhood immunisation schedule (which may positively affect uptake of other childhood vaccines)	10,11
Respiratory syncytial virus (RSV)	Recommendation of use of long-acting monoclonal antibodies (mAb) in newborns and infants; no recommendation for RSV vaccines in pregnancy	2024	• High incidence and burden of disease in Germany • Mathematical modelling and health economic evaluation^b^ of different immunisation strategies (maternal vaccination vs. long-acting mAbs in all infants vs. the status quo of using short-acting mAbs in high-risk infants), with results favouring long-acting mAbs • Implementation considerations regarding impact of an additional vaccine on uptake of other vaccines during pregnancy • Limited data (e.g., vaccine safety) and experience with maternal RSV vaccination in other countries at time of decision-making	12,13
Herpes zoster (HZ)	Recommendation of routine vaccination of adults aged 60+ with an adjuvanted subunit vaccine (not previously recommended)	2018	• Local disease burden (incidence of HZ and postherpetic neuralgia by age) • Mathematical modelling and health economic analysis^b^ to define efficient target population • Values and preferences regarding outcomes • Goal of the immunisation strategy to prevent HZ-related complications and improve quality of life	14,15
Rotavirus (RV)	Recommendation of routine oral vaccination for infants from 6 weeks	2013	• Local disease burden, especially RV-associated hospitalisations • Health economic analysis^b^ to determine cost-effectiveness of the vaccination • Values and preferences regarding outcomes • Goal of the immunisation strategy to reduce morbidity (as RV-associated deaths are very rare in Germany)	16,17

HZ: Herpes zoster; mAb: monoclonal antibody; RSV: respiratory syncytial virus; RV: rotavirus.

a The drivers listed are illustrative of key considerations and do not represent an exhaustive account of STIKO's deliberations.

b In Germany, health economic assessments of vaccines do not contain a cost-effectiveness threshold and do not constitute a decisive criterion.

## Implications for international collaboration, joint assessments, and harmonisation

National immunisation programmes share a common core of foundational vaccines. Routine vaccinations against diphtheria, tetanus, pertussis, poliomyelitis or measles, for example, should and are recommended consistently across European national programmes. However, differences in national vaccination schedules may exist in the number of additional vaccines beyond this core, as well as regarding exact age and intervals when vaccines should be administered, number of doses, or selection of specific target groups.^7^ Such differences do not necessarily imply differences in scientific standards and should not be read as inconsistencies, but as the appropriate outcome of tailored, evidence-based decision-making, reflecting the role of country-specific evidence and considerations in immunisation recommendations. NITAGs play a central role in this contextualisation because of their legal mandate in many countries, detailed knowledge of the national health system and immunisation programme, and specific expertise in assessing programmatic feasibility and the full value of vaccines to their populations. NITAGs not only consider different country-specific evidence but may also weight individual criteria differently depending on national context, priorities, and societal values, and this differential weighting is an integral part of the recommendation process.

The increasing interest in conducting joint evidence assessments, comparing national immunisation schedules, or harmonising immunisation recommendations between countries must be viewed in light of context-specific considerations. Joint evidence assessments or JCAs of new vaccines can usefully deliver a robust common synthesis of context-free evidence (safety, efficacy, and effectiveness) and reduce the workload in countries. However, they offer a narrowly focused view and cannot deliver the country-specific synthesis of data and considerations that ultimately drives national recommendations — including local disease burden, health economic context, programmatic feasibility, and societal values — which cannot be resolved at regional or global level. Joint evidence assessments, therefore, may not automatically lead to quicker vaccine introductions and uniform vaccination schedules across Europe. Similarly, the development of EU-level vaccination recommendation will have limits since they cannot integrate and sufficiently reflect country-specific considerations of 27 EU member states. While equitable population access to vaccines is an important aspect in immunisation policymaking, country context cannot be ignored. The assessment of country-specific evidence, especially the collection of robust epidemiological intelligence and synthesis of all factors relevant to immunisation recommendation decision-making, and the development of country-specific mathematical dynamic transmission models (where appropriate), remain essential and take time and effort.

The free movement of people and goods within the EU has been highlighted as a particular consideration for vaccine policy.^18^ While cross-border mobility supports the case for coordinated infrastructure such as shared surveillance, a European vaccination card, or shared information on national vaccination schedules (e.g., via ECDC’s vaccine scheduler),^7^ pre-travel consultations and routine healthcare interactions in the destination country (especially for long-term residents) typically allow individuals to receive appropriate immunisation advice and catch-up vaccinations where needed. Therefore, free movement does not, in itself, constitute a strong rationale for harmonising national immunisation schedules, especially when local disease burden, costs or health systems differ.

Although differences in recommendations between countries are legitimate, recommendation-making processes should be transparently documented. In Germany, STIKO publishes a detailed scientific rationale for every recommendation, documenting the evidence considered, the weighting of context-free and context-specific considerations, and the reasoning that led to the final recommendation. Such transparent documentation of the rationale behind recommendations is essential to maintain trust and facilitate understanding among stakeholders and the public. It also enables comparison and learning between NITAGs and contributes to consistent scientific standards across countries despite differences in the resulting recommendations.

As the number of new vaccines grows and budgets for national immunisation programmes are limited, the importance of country-driven processes will only increase. For this reason, WHO’s SAGE has called for greater country ownership and strengthening of NITAGs to ensure that immunisation programmes deliver maximum population health impact.^19^ International collaboration in immunisation is most productive when it supports national processes and builds capacity. When guidance on immunisation policy is developed at supra-national level, it should leave sufficient flexibility and options to allow country contextualisation, to avoid unnecessary pressure and need for justifications, and to strengthen country ownership and responsibility.

## Contributors

All authors conceptualised this manuscript. RS wrote the initial draft. All authors contributed significantly to the revisions and final version of the article, which is approved by all authors.

## Declaration of interests

The authors declare no competing interests.
